# The tick endosymbiont *Candidatus* Midichloria mitochondrii and selenoproteins are essential for the growth of *Rickettsia parkeri* in the Gulf Coast tick vector

**DOI:** 10.1186/s40168-018-0524-2

**Published:** 2018-08-13

**Authors:** Khemraj Budachetri, Deepak Kumar, Gary Crispell, Christine Beck, Gregory Dasch, Shahid Karim

**Affiliations:** 1 Department of Biological Sciences, University of Southern Mississippi, Hattiesburg, MS 39406 USA; 2 Delta State University, Cleveland, MS 38733 USA; 3 Rickettsial Zoonoses Branch, Centers for Disease Control, Atlanta, GA 30329 USA

**Keywords:** *Rickettsia parkeri*, Endosymbionts, Ticks, Selenogenes, Pathogen, Colonization

## Abstract

**Background:**

Pathogen colonization inside tick tissues is a significant aspect of the overall competence of a vector. *Amblyomma maculatum* is a competent vector of the spotted fever group rickettsiae, *Rickettsia parkeri.* When *R. parkeri* colonizes its tick host, it has the opportunity to dynamically interact with not just its host but with the endosymbionts living within it, and this enables it to modulate the tick’s defenses by regulating tick gene expression. The microbiome in *A. maculatum* is dominated by two endosymbiont microbes: a *Francisella*-like endosymbiont (FLE) and *Candidatus* Midichloria mitochondrii (CMM). A range of selenium-containing proteins (selenoproteins) in *A. maculatum* ticks protects them from oxidative stress during blood feeding and pathogen infections. Here, we investigated rickettsial multiplication in the presence of tick endosymbionts and characterized the functional significance of selenoproteins during *R. parkeri* replication in the tick.

**Results:**

FLE and CMM were quantified throughout the tick life stages by quantitative PCR in *R. parkeri*-infected and uninfected ticks. *R. parkeri* infection was found to decrease the FLE numbers but CMM thrived across the tick life cycle. Our qRT-PCR analysis indicated that the transcripts of genes with functions related to redox (selenogenes) were upregulated in ticks infected with *R. parkeri*. Three differentially expressed proteins, selenoprotein M, selenoprotein O, and selenoprotein S were silenced to examine their functional significance during rickettsial replication within the tick tissues. Gene silencing of the target genes was found to impair *R. parkeri* colonization in the tick vector. Knockdown of the selenogenes triggered a compensatory response from other selenogenes, as observed by changes in gene expression, but oxidative stress levels and endoplasmic reticulum stress inside the ticks were also found to have heightened.

**Conclusions:**

This study illustrates the potential of this new research model for augmenting our understanding of the pathogen interactions occurring within tick hosts and the important roles that symbionts and various tick factors play in regulating pathogen growth.

**Electronic supplementary material:**

The online version of this article (10.1186/s40168-018-0524-2) contains supplementary material, which is available to authorized users.

## Background

Ticks are blood-feeding ectoparasites of both humans and animals and are important from a public health perspective because they serve as competent vectors of various disease-causing infectious agents. Many tick-borne pathogens are equipped to infect various tick organs where they can multiply. Infection of the salivary glands enables tick pathogens to readily infect vertebrate hosts upon tick feeding. The spotted fever group rickettsial (SFGR) agent, *Rickettsia parkeri*, is maintained in tick populations through transstadial (between life stage molts) and transovarial or vertical transmission (deposition into eggs for next-generation pathogen development) [1]. *A. maculatum*, the Gulf Coast tick, is an arthropod vector with increasing public health significance because of its role as the primary vector of *R. parkeri* in the USA [2]. Rickettsial diseases are caused by obligate intracellular Gram-negative bacteria, and these organisms infect humans on all continents except Antarctica [3–5]. In modern times, the rate and ease of global movement has increased the risk of transporting ticks and tick-borne diseases that may have previously been restricted to one region.

The microorganisms that occupy a tick vector are collectively called the tick microbiome; however, the collection of commensal, symbiotic, and pathogenic microbes associated with ticks are more specifically termed the “pathobiome.” Although the inclusion of all three microbial types appears to be counterintuitive at first sight, it is possible that microbes living in association with pathogens within ticks can positively influence pathogen transmission. For instance, rickettsial endosymbionts are thought to alter the transmission of other rickettsial pathogens, as shown by the inverse relationship between the infection prevalence of *Rickettsia rickettsii* (pathogen) and *R. peacockii* (symbiont) in the Rocky Mountain wood tick *Dermacentor andersoni* [6, 7]. Likewise, the presence of *Coxiell*a-related symbionts in the salivary glands of *A. americanum* ticks has been proposed to impair the transmission of *Ehrlichia chaffeensis* [8]. In addition to symbionts, ticks maintain a natural bacterial flora predominantly composed of *Proteobacteria*, *Firmicutes*, and *Bacteroides* phyla [9–14], which have also been implicated in pathogen maintenance interference in the tick. For example, when *Ixodes scapularis* ticks are hatched and raised in a sterile environment, their microbiota is altered such that they experience impaired gut integrity and reduced colonization ability towards *Borrelia burgdorferi* [15]. As seen with other arthropod vectors, altering the tick microbiome may also result in a modulated type of immune response that can interfere with pathogen survival and infection [16].

A dynamic interaction happens between tick vectors and their associated disease-causing agents, and this has been referred to as a continuous “*bellum omnium contra omes*” or war of all against all [17]. An unavoidable interaction between a pathogen and the obligate symbiont(s) in a vector occurs during colonization and transmission. However, understanding about the interactions between rickettsial endosymbionts and pathogenic bacteria in ticks and how they influence each other is limited. There are few published reports on the roles played by symbionts in ticks and whether these bacteria have an impact on tick proliferation or transmission [18, 19]. The symbionts commonly associated with hard ticks belong to *Rickettsia, Francisella, Coxiella, Wolbachia*, and *Candidatus* Midichloria genera [20]. *Francisella*-like endosymbionts (FLE), which have been detected in many ticks [21–23], are γ-proteobacterial symbionts and are related to the bacterium that causes tularemia, *Francisella tularensis* [24]. Genetically distinct FLEs have been reported in *D. variabilis* and *D. andersoni* [25] and across the tick’s developmental stages [26]. Gerhart et al. [27] hypothesized that pathogenic *F. tularensis* was capable of transforming into symbiotic FLE in ticks. *Candidatus* Midichloria mitochondrii (CMM), an α-proteobacterial symbiont first detected in *I. ricinus*, has a unique intramitochondrial lifestyle [28]. Based on phylogenetic and statistical studies of the 16S rRNA sequences from *Midichloria* and “similar organisms,” CMM is proposed to belong to a novel family known as “*Candidatus* Midichloriaceae” [29] and is widespread in various ixodid ticks [30]. However, our understanding of the interactions between endosymbionts (FLE, CMM) and pathogenic bacteria (*R. parkeri*) in tick tissues and how they influence each other remains limited.

In the absence of preventive measures, the increasing number of tick-borne diseases poses a significant threat to public health. To survive, ticks must maintain homeostasis (stable equilibrium maintained by physiological processes) and obtain gigantic blood meals of up to 100 times their unfed weight. Selenium (Se) is an essential trace element that is incorporated as selenocysteine (Sec) into selenoproteins (SELENO), many of which form an essential line of defense against oxidative stress damage [31]. These proteins are also responsible for myriad other functions including Se transport, protein folding, and endoplasmic reticulum-associated degradation (ERAD). The endoplasmic reticulum (ER) is involved in intracellular signaling, protein synthesis and protein folding, glycosylation, and secretion of saliva via the exocytotic pathway. Tick saliva composition, as revealed by our sialotranscriptome (from the Greek, sialo means saliva), indicated the presence of over 5000 putative secreted peptides representative of dozens of protein families [32, 33]. Protein folding is dependent on the oxidation of disulfide bridges via reactive oxygen species (ROS). A heightened oxidative environment can impair protein folding, leading to the accumulation of unfolded or misfolded proteins and ultimately ER stress. ER homeostasis can be disrupted by a variety of insults such as the accumulation of misfolded proteins, elevated levels of ROS, pathogen infections, and abnormalities in Ca^+ 2^ signaling. These disturbances are able to trigger the unfolded-protein response (UPR), a protective counter-measure that acts to reestablish homeostatic balance and promote survival by increasing the production of the chaperones involved in protein folding or by inhibiting global translation and eliminating chronically misfolded proteins. Proteins that fail to properly fold are eliminated via ERAD. ER-resident selenoproteins play a critical role in modulating oxidative and ER stress during prolonged tick feeding on the host. We have discovered multiple factors involved in the synthesis of the tick selenoproteome (a full set of novel selenoproteins in ticks), including a novel eukaryotic elongation factor (eEFSec), a novel SECIS-binding protein (SBP2), and Sec-tRNA^Sec^ [31–34]. The serendipitous RNA-Seq findings from our experimental gene silencing of *eEFSec* indicated that dramatic changes in the expression patterns of the transcripts encoding secreted salivary proteins had occurred [34]. Our further studies revealed that selenoproteins and antioxidants participate in SFGR colonization within the tick vector and in their vertical transmission to the next generation [31, 34–38]. Interestingly, *I. scapularis* Salp25D (a glutathione peroxidase) and *D. variabilis* SELENOM confer a survival advantage on *B. burgdorferi* [39] and *Anaplasma marginale* [40].

In the present study, we used two approaches to gain a better understanding of the replication and physiology of *A. maculatum* *R. parkeri* infected (Rp^+^) and R. parkeri-free (Rp^−^) colonies isolated from field collection and continuously propagated at The University of Southern Mississippi, USA. First, we examined the potential interplay between pathogenic *Rickettsia* (*R. parkeri*) and the dominant non-pathogenic tick symbionts by quantifying the FLE and CMM symbiont loads with or without rickettsial infections. Second, we investigated the differential gene expression of specific tick selenogenes in *R. parkeri*-infected tissues. Third, we utilized an RNA interference approach to deplete the expression of differentially regulated *SELENOM*, *SELENOO*, and *SELENOS* selenogenes and assess their functional significance in pathobiome maintenance in the tick vector. Overall, we have shown that *R. parkeri* replication success is correlated with the quantity of CMM present in the tick at the expense of FLE in the tick, and that selenogenes play important roles in tick–pathogen interactions.

## Results

### Quantitation of *R. parkeri*, FLE, and CMM across the tick life cycle

*R. parkeri*-infected (Rp^+^) and *R. parkeri*-free (Rp^−^) *A. maculatum* tick colonies were established and maintained in the laboratory for studying the dynamics of symbiont–*Rickettsia* interactions. Our previous microbiome analysis of *A. maculatum* identified FLE and *R. parkeri* in *A. maculatum* ticks [9]. Recently, our Illumina-sequenced sialotranscriptome work has also detected significant numbers of reads from CMM in *A. maculatum* (Shahid Karim, unpublished results).

**Fig. 1:**
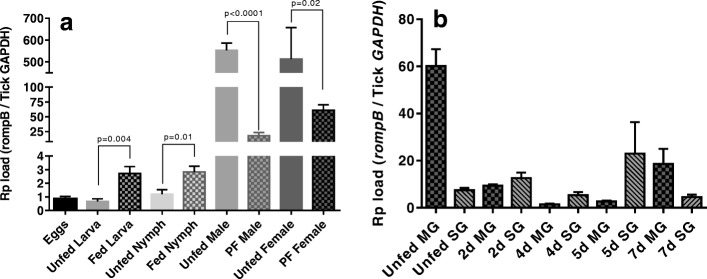
Transovarial and transstadial maintenance of *R. parkeri* loads during the life stages of *A. maculatum* ticks. **a** Estimated *R. parkeri* load in immature and mature developmental stages of the tick, including the eggs, larva (unfed and blood-fed), nymphs (unfed and blood-fed), and adult males and females (unfed and partially blood-fed). **b** Time-dependent and tissue-specific *R. parkeri* load estimated in tick midgut and salivary gland tissues across different time points during tick infestation on sheep. The *R. parkeri*-infected ticks were infested on sheep and 5–7 ticks were removed from the host on days 2, 4, 5, and 7 post-infestation. Within 2 h of removal from the host, the individual ticks were dissected and their midgut tissues and salivary glands removed. The tissues from individual ticks were stored in RNAlater, RNA was extracted, and qRT-PCR was performed using *rompB*-specific primers. *GAPDH* primers were used to estimate the number of *R. parkeri* copies per tick *GAPDH*. At least three biological replicates were used in these experiments

**Fig. 2:**
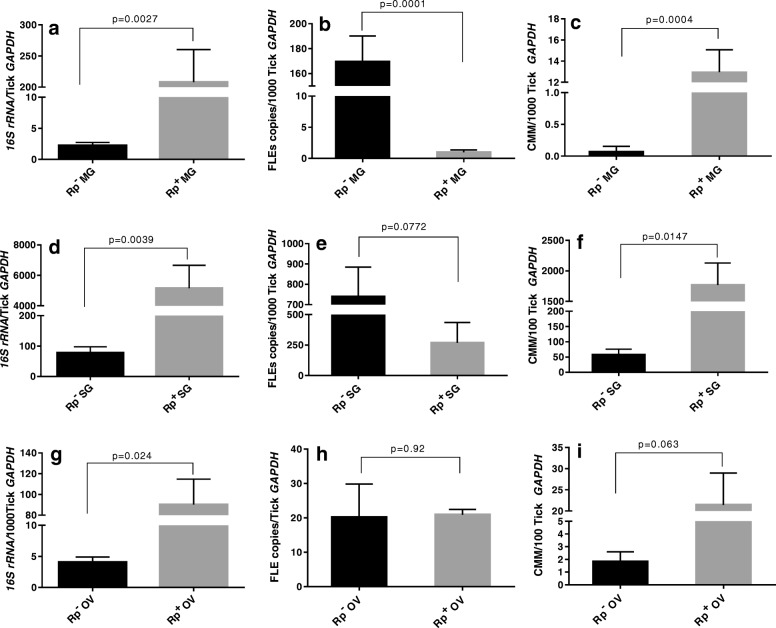
Total bacterial load, *Francisella-*like endosymbiont (FLE) load, and *Candidatus* Midichloria mitochondrii (CMM) load in tick tissues (midguts, salivary glands, ovaries) from *R. parkeri-*infected (Rp^+^) and uninfected (Rp^−^) *A. maculatum* female ticks. The ticks from both Rp^+^ and Rp^−^ colonies were infested on two separate sheep for blood feeding and 5–15 ticks were removed from the host on day 5 post-infestation. Within 2 h of tick removal from the hosts, the ticks were dissected to isolate their tissues (midgut, salivary glands, and ovarian tissues) and each midgut or salivary gland was individually placed in separate vials and five tick ovaries were pooled in a vial and stored in RNAlater before RNA extraction and cDNA synthesis. Total bacterial loads and FLE and CMM copies/ tick were estimated by qPCR with reference to *GAPDH* in the tick midgut tissues (**a**, **b**, **c**), salivary gland tissues (**d**, **e**, **f**) and ovaries (**g**, **h**, **i**) in the Rp^+^ ticks (black bars) and the Rp^−^ ticks (gray bars). Rp, *R. parkeri*; OV, ovarian tissues; Mg, midguts; Sg, salivary glands

**Fig. 3:**
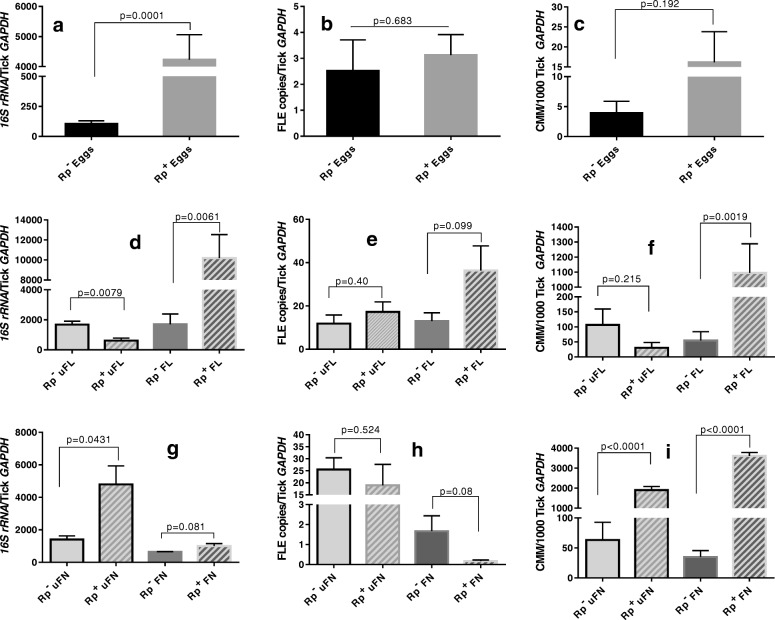
Total bacterial load (BL), *Francisella*-like endosymbiont (FLE) load, and *Candidatus* Midichloria mitochondrii (CMM) load in eggs, unfed larva, blood-fed larva, unfed nymphs, and blood-fed nymphs. *R. parkeri*-infected (Rp^+^) and uninfected (Rp^−^) *A. maculatum* gravid females were allowed to oviposit, and approximately 25 days after egg incubation, about 20 mg of the egg masses were sampled from three gravid females separately. When the remaining eggs hatched into larvae, the unfed larvae were allowed to feed on the blood of an individual hamster until repletion occurred. The dropped-off larvae were collected and three from each Rp^+^ and Rp^−^ group were stored in RNAlater. The remaining engorged larvae were incubated for 30 days at which point they molted into nymphal ticks, and the unfed nymphs were blood-fed until repletion. Three engorged nymphs from the Rp^+^ and Rp^−^ groups were stored in RNAlater. Three biological replicates were used for all the treatments. The total bacterial load, FLE, and CMM copies/ tick *GAPDH* in Rp^+^ and Rp^−^ ticks were determined using gene-specific primers (Additional file 5: Table S1). Total bacterial load, FLE and CMM loads in eggs (**a**, **b**, **c**), larva (**d**, **e**, **f**), and nymphal ticks (**g**, **h**, **i**) are shown. uFL, unfed larva; FL, fed larva; uFN, unfed nymph; FN, fed nymphs

We observed that *R. parkeri*, together with the FLE and CMM intracellular symbionts, are transovarially and transstadially transmitted in Rp^+^ *A. maculatum* ticks (Figs. 1, 2, and 3). We also found that the relative concentrations of *R. parkeri* increased across the tick developmental stages from eggs to adults (Fig. 1). The infected eggs that hatched into infected larvae contained similar concentrations of *R. parkeri*, but the *R. parkeri* load significantly increased (> 3.5 fold) with the blood meal in the fed larvae (*p* = 0.0041). *R. parkeri* was transstadially transmitted to the nymphal stages and after the nymphal blood meal the *R. parkeri* load significantly increased (> 2 fold) (*p* = 0.01). The concentrations of *R. parkeri* in molted adult males and females were only slightly greater than in the fed nymphs. In the adult ticks, the *R. parkeri* load decreased in the midgut tissues and the salivary glands upon prolonged blood feeding on the host (Fig. 1b).

The relative concentrations of FLE and CMM in the total bacterial load were estimated in the Rp^+^ and Rp^−^ ticks to assess the potential interplay that may occur during bacterial replication among *R. parkeri,* FLE, and CMM (Figs. 2 and 3) across the immature and mature developmental stages of the ticks. The total bacterial concentration was significantly reduced in the Rp^−^ eggs compared with the Rp^+^ eggs (Fig. 2a–c). In the unfed Rp^+^ larvae, the total bacterial concentration was lower than in the unfed Rp^−^ ticks, but a significant effect was not observed for FLE or CMM (Fig. 2d–f). However, the larval blood meal in the Rp^+^ ticks greatly enhanced the total bacterial load and the FLE and CMM concentrations relative to those in the Rp^−^ ticks (Fig. 3d–f). The outcome of this experiment in the nymphs did not parallel those for the larval stage (Fig. 3g–i). Indeed, the Rp^+^ ticks had reduced FLE loads in the fed and unfed nymphs as compared with the Rp^−^ ticks. Similarly, the total bacterial and CMM loads were seen to increase in the Rp^+^ nymphs compared with the Rp^−^ nymphs regardless of feeding status (Fig. 3g–i).

The presence of an *R. parkeri* infection increased the total bacterial load in the Rp^+^-infected female gut tissues, salivary glands, and ovarian tissues (Fig. 2). The total bacterial load [10] in naïve ticks (from the Oklahoma State Tick Rearing Facility), along with the FLE levels, were found to decrease rapidly in both tick midgut and salivary gland tissues after the blood meal in the Rp^−^ female adults (Additional file 1: Figure S1), whereas the CMM level remained fairly constant in the midgut tissues over the course of the blood meal but decreased rapidly in the salivary glands after the blood meal (Additional file 1: Figure S1).

### *Rickettsia parkeri* infection differentially regulates tick selenogene expression

**Fig. 4:**
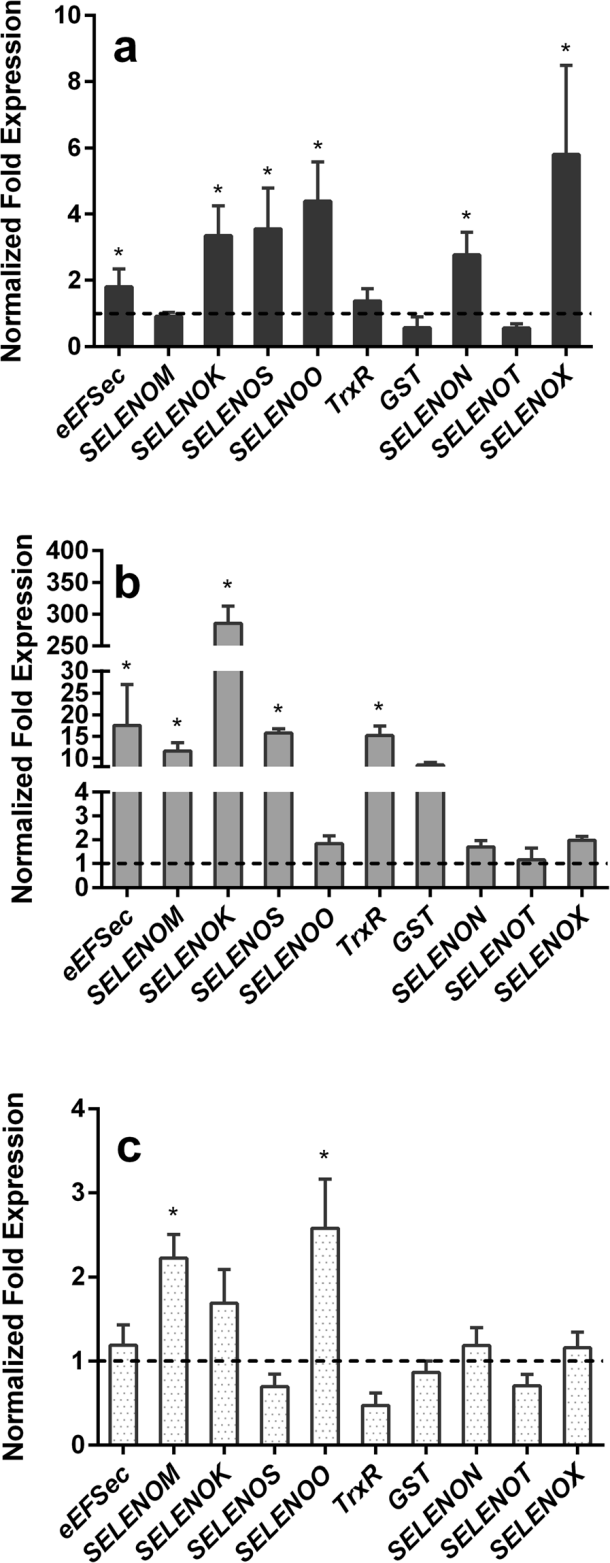
Differentially expressed tick selenogenes in *R. parkeri*-infected (Rp^+^) adult female ticks on day 5 after feeding. The Rp^+^ and *R. parkeri*-free adult female ticks that fed on sheep (in Fig. 1) were removed from the host on day 5 post-infestation and then dissected for tissue collection (midguts, salivary glands, and ovaries). Tick midguts and salivary glands isolated from single ticks and ovarian tissues were pooled from five individually-dissected Rp^+^ and Rp^−^ ticks. Quantitative reverse transcriptase PCR (qRT-PCR) was used to determine the transcriptional expression levels of the tick selenogenes. Differential gene expression was estimated in **a** tick midguts, **b** salivary glands, and **c** ovarian tissues. The expression levels in the Rp^−^ tick tissues were set to 1, as represented by dashed lines. eEFSec: selenocysteine elongation factor, *SELENOM*: selenoprotein M, *SELENOK*: selenoprotein K, *SELENOS*: selenoprotein S, *SELENOO*: selenoprotein O, *TrxR*: thioredoxin reductase, *GST*: glutathione S-transferase, *SELENON*: selenoprotein N, *SELENOX*: selenoprotein X, *SELENOT*: selenoprotein T

We assessed the differential expression of ten tick selenoprotein genes in Rp^+^ and Rp^−^ adult female ticks at 5 days post-infestation (dpi) of the ticks by quantitative reverse transcriptase (qRT)-PCR (Fig. 4). Six genes (*eEFSec*, *SELENOK*, *SELENOS*, *SELENOO*, *SELENON*, and *SELENOX*) were significantly upregulated (*p* < 0.05) in the midgut tissues, and five selenogenes (*eEFSec*, *SELENOM*, *SELENOK*, *SELENOS*, *TrxR*) were significantly upregulated (*p* < 0.05) in the salivary glands, while the levels of *SELENOO*, *SELENON*, and *SELENOX* remained unchanged (Fig. 4). Interestingly, only *SELENOM* and *SELENOO* genes were significantly upregulated in the Rp^+^-infected ovarian tissues, but *SELENOT* was not differentially regulated in any of the tested tissues (Fig. 4). Based on these results, the following three differentially expressed selenogenes encoding the following proteins were selected to determine their functional significance in tick blood feeding and pathogen colonization within the tick vector: endoplasmic reticulum-resident Selenoprotein M (gene, *SELENOM*), Selenoprotein S (gene, *SELENOS*), and mitochondrial resident Selenoprotein O (gene, *SELENOO*).

Temporal- and tissue-specific *SELENOO* and *SELENOS* transcript levels were assessed in the unfed and partially-fed midguts and salivary glands in from the Rp^−^ female adults (Additional file 2: Figure S2). Interestingly, the gene expression level of SELENOO in the midgut tissues was upregulated 2–2.5-fold, while the salivary glands showed decreased transcriptional expression from 2 to 8 dpi (Additional file 2: Figure S2a). In contrast, the *SELENOS* transcript level was fourfold upregulated during the early phase of tick feeding compared with the tissue levels in the unfed ticks (Additional file 2: Figure S2). *SELENOS* expression remained unchanged in the midgut tissues from 2 to 8 dpi (Additional file 2: Figure S2b). The transcriptional activity of *SELENOM* has been reported to gradually increase up to 2 dpi but diminish thereafter in both tissue types [35].

### Impact of selenoprotein gene silencing on tick physiology

**Fig. 5:**
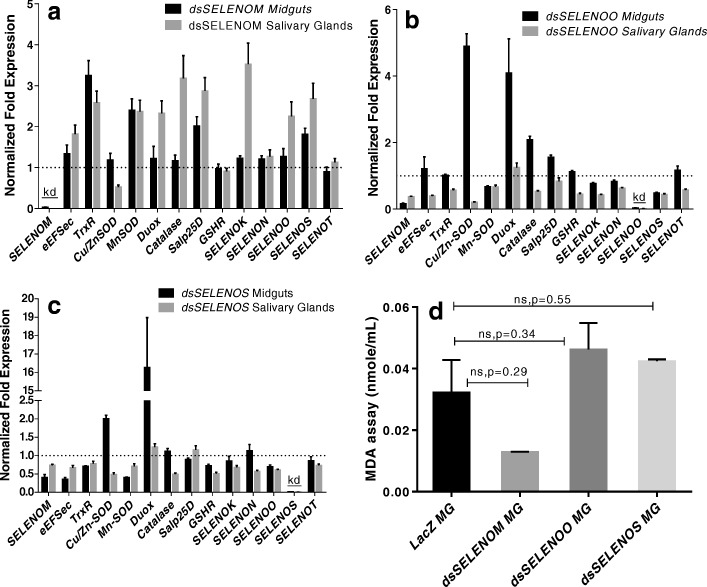
Functional characterization of tick selenoprotein gene knockdowns in *R. parkeri-*infected (Rp^+^) *A. maculatum*. A dsRNA-based silencing assay was performed for **a** *SELENOM*, **b** *SELENOO*, and **c** *SELENOS* in Rp^+^ ticks, and the compensatory expression levels of tick antioxidants and selenoproteins were estimated. The dsRNA specific for each selenogene (*SELENOM*, *SELENOO*, and *SELENOS*) was synthesized to include the addition of a T7 RNA polymerase binding site as the flanking sequence in the individual selenogene PCR amplicons from the dsRNA (Additional file 5: Table S1) and the in vitro RNA synthesis (which utilized the HiScribe™ T7 High Yield RNA synthesis kit, New England Biolabs). The dsRNA synthesized for each selenogene, along with irrelevant dsLacZ, were microinjected into 25–30 Rp^+^ ticks or 25–30 *R. parkeri*-free ticks (Additional file 3: Figure S3). The microinjected ticks were allowed to replete on sheep, and 5–10 ticks were removed from them to study the impact on gene silencing and the impact on *Rickettsia parkeri* and other symbionts (Figs. 6 and 7) on day 5 post-infestation. In each selenogene-silenced tick tissue (**a** *SELENOM*, **b** *SELENOO*, and **c** *SELENOS*), the transcript levels of a panel of selenogenes (*SELENOM*, *SELENOO*, and *SELENOS*, along with *eEFSec*, *TrxR*, *SELENOK*, *SELENON*, *SELENOT*) and redox genes (*Cu/Zn*-*SOD*, *Mn-SOD*, *Duox*, *Catalase*, *GSHR*, *Salp25D*) were measured. The transcript level for each gene in the control tissues was normalized to 1 for reference and is represented here as a dashed line. Tick *GAPDH* was used as a reference gene for normalizing the qRT-PCR results. **d** Oxidative stress in the selenogene-silenced tick midguts and control (dsLacZ) midguts was estimated using a malondialdehyde assay. KD, knockdown

**Fig. 6:**
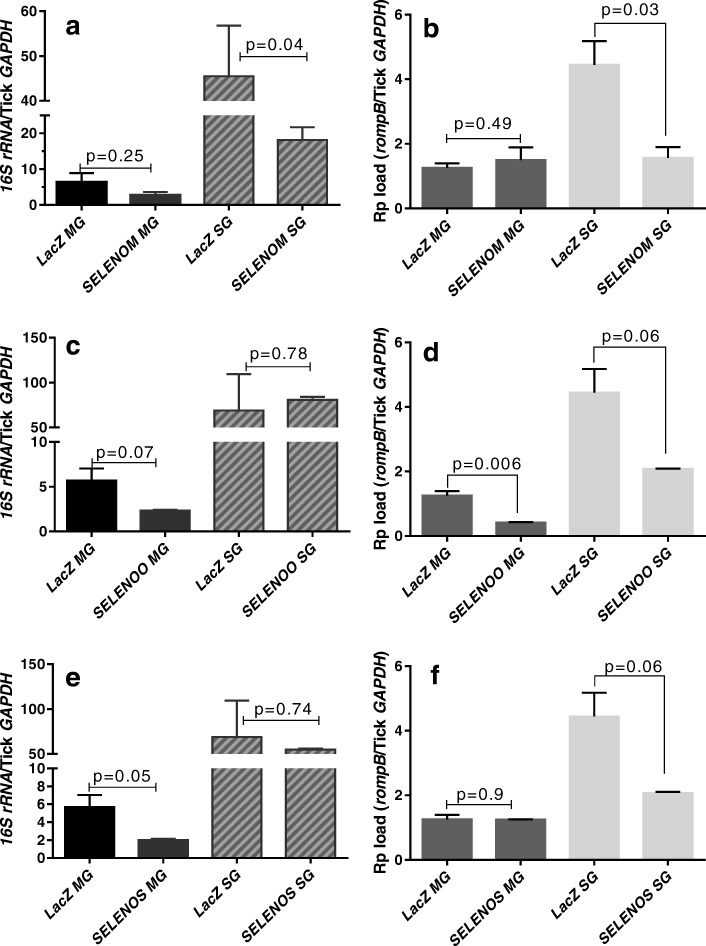
The impact of tick selenoprotein gene silencing on total bacterial and *R. parkeri* loads in the tick. The selenogene-silenced tick tissues (from Fig. 5) 5 days after microinjections of dsRNA–*SELENOM,* dsRNA–*SELENOO*, or dsRNA–*SELENOS* were used to estimate the total bacterial load (BL) and *R. parkeri* (Rp) load in the *SELENOM*- (**a**, **b**), *SELENOO*- (**c**, **d**), and *SELENOS*-silenced ticks (**e**, **f**). The qRT-PCR assay described in Figs. 1 and 2 was used to estimate the total bacterial load and Rp load per tick GAPDH. The *p* value is provided to compare statistical significance between the selenogene-silenced ticks and the control ticks. A *p* value of < 0.05 was considered statistically significant

We employed the RNAi approach to silence *SELENOM*, *SELENOO*, and *SELENOS* tick selenoproteins in Rp^−^ and Rp^+^ *A. maculatum* ticks. The results obtained for the silencing of selenoprotein genes in Rp^−^ ticks has been published for *SELENOM* [35] and is shown here as an Additional file 3: Figure S3. To examine the roles of each of the three selenogenes in *R. parkeri*-infected ticks, transcripts from the three selenogenes were individually depleted using RNAi (Fig. 5), and the effects of the depletion on *R. parkeri* replication were determined (Fig. 6). Silencing of these selenogenes did not negatively impact blood feeding in the ticks (data not shown). We achieved > 90% depletion of the transcriptional expression of all selenogenes under investigation in the uninfected ticks (except for *SELENOO* in Rp^−^ ticks) (Additional file 3: Figure S3). Despite the effectiveness of the *SELENOM* knockdowns, the transcriptional expression levels of *TrxR*, *Mn-SOD*, *Duox*, *Cat*, *Salp25D*, *SELENOK*, *SELENOO*, and *SELENOS* were 2–3-fold upregulated in the partially blood-fed salivary glands of the Rp^+^ ticks (Fig. 5a). Moreover, *TrxR*, *Mn-SOD*, and *Salp25D* showed 2–3-fold increases in their expression levels in the midgut tissues (Fig. 5a). *SELENOO* depletion resulted in a 2–5-fold upregulation of *Catalase*, *Cu/Zn-SOD*, and Duox in the midgut tissues, whereas the other selenogenes were down-regulated in the salivary gland tissues (Fig. 5b). The *SELENOS* knockdown produced a 2–16-fold upregulation of *Cu/Zn-SOD* and *Duox* in the midgut tissues, while the transcript levels of the other tested genes remained unchanged or were downregulated in the salivary glands tissues (Fig. 5c). Tick *SELENOM*, *SELENOO*, and *SELENOS* silencing had no significant impact on the total oxidative stress levels, as estimated by the MDA lipid peroxidation method (Fig. 5d). The impact of knocking down these selenoproteins on ER stress was estimated by measuring the transcriptional expression of the unfolded protein response sensor genes, *IRE1* and *AFT6* (Table 1). The *SELENOO* and *SELENOS* knockdowns resulted in upregulation of both *ATF6* and *IRE1* sensor genes in the Rp^+^ tick midgut but not in the Rp^−^ ticks (Additional file 4: Figure S4a), nonetheless opposite in *SELENOM* (Additional file 4: Figure S4b).

**Table 1:** Summary of results

	Parameters	*Rickettsia parkeri* (Rp-) ticks	*Rickettsia parkeri* (Rp+) ticks
*Rickettsia parkeri* and symbionts dynamics	*R. parkeri* load (Rp)	No. *R. parkeri*	Increases load with blood meal in immature ticks. Decreases load along the blood meal in adult (female) tissues.
	*Candidatus* Midichloria mitochondrii (CMM)	Present across the tick life cycle. Blood meal reduces the abundances.	Proliferates with *Rp* infections.
	*Francisella*-like endosymbiont (FLE)	Present across the tick life cycle. Blood meal reduces the abundances.	May get displaced with *Rp* infections.
	Total bacterial load (BL)	Blood meal reduces the abundances.	Increase with Rp infections.
Transcriptional expressions of selenoproteins	Midgut tissues	*SelO* and *SelS* constantly express while *SelM* is cyclical (gradually peaking up and then decreases).	*SELENOK*, *SELENOO*, *SELENOS*, *SELENON*, and *SELENOX* upregulated
	Salivary glands	Spiked at unfed (*SelO*) and spiked at early feeding and later diminishes (*SelS*) while *SelM* cyclical.	*eEFSec*, *SELENOM*, *SELENOK*, *SELENOS*, *TrxR*, and *GST* upregulated
	Ovarian tissues	nd	*SELENOM* and *SELENOO* upregulated
Knockdown of selenoproteins	ΔSelM	–	SG: depleted Rp, BL, and FLE
	ΔSelO	–	MG: depleted Rp, BL, and CMM
	ΔSelS	–	MG: depleted BL, FLE SG: depleted CMM

Knockdown of SELENOM or SELENOO or SELENOS by RNAi method. Nd: not determined across the blood meal

*MG* midguts, *SG* salivary glands, *Rp Rickettsia parkeri*, *CMM Candidatus* Midichloria mitochondrii, *FLE Francisella*-like endosymbionts, ΔSelM or SelO or SelS

### Impact of selenoprotein silencing on total bacterial load and *R. parkeri* replication

Knocking-down *SELENOM* resulted in a significant decrease in the *R. parkeri* concentration and the total bacterial load in the salivary gland tissues, but not in the midgut tissues after 5 dpi in the female ticks (Fig. 6a, b). The total bacterial load decreased in the midguts upon *SELENOO* knockdown, but the result was not statistically significant (*p* = 0.07), and the bacterial load in the salivary glands remained unaffected (Fig. 6c). The *SELENOO* knockdown depleted *R. parkeri* (*p* = 0.0062) in the midgut tissues, but the *R. parkeri* levels remained unchanged in the salivary glands (*p* = 0.06) (Fig. 6d). Finally, the *SELENOS* gene silencing depleted the total bacterial load in the midgut but not in the salivary gland tissues (Fig. 6e). Similarly, the *SELENOS* knockdown did not alter the *R. parkeri* load in the midgut tissues (*p* = 0.97), unlike in the salivary gland tissues where it was reduced, but not significantly so (*p* = 0.06) (Fig. 6f).

### Quantification of tick symbionts upon selenogene silencing

**Fig. 7:**
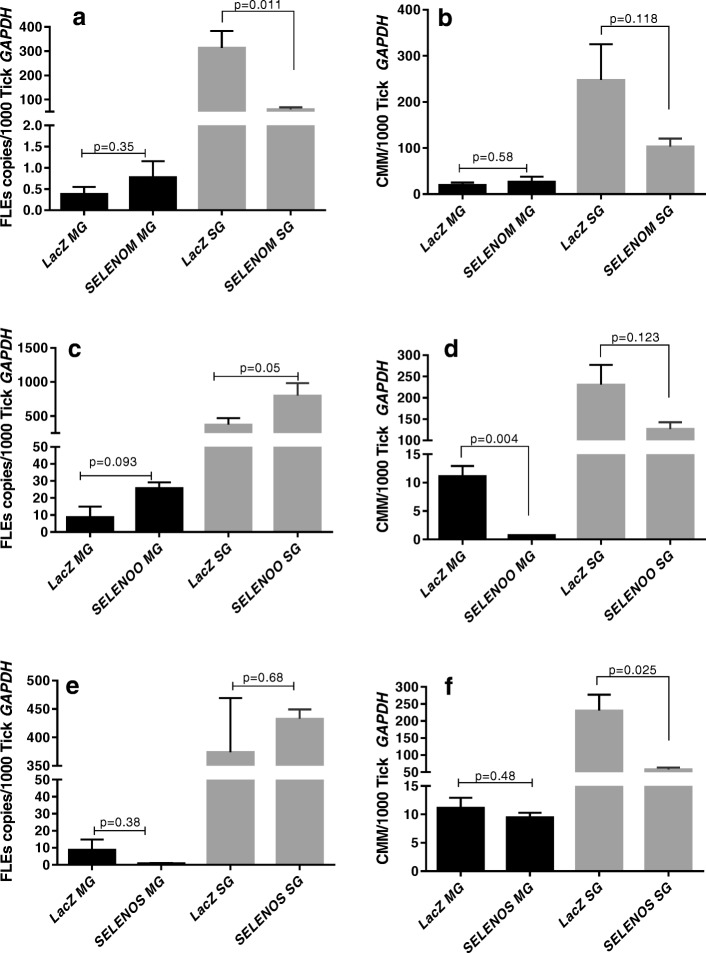
The impact of selenoprotein silencing on tick symbionts in *R. parkeri* (Rp^+^)-infected *A. maculatum*. The selenogene-silenced tick tissues (from Fig. 5) on day 5 post-microinjection of dsRNA–*SELENOM*, dsRNA–*SELENOO*, or dsRNA–*SELENOS* were used to determine the total bacterial load (BL) and Rp load in *SELENOM*- (**a**, **b**), *SELENOO*- (**c**, **d**), and *SELENOS*-silenced ticks (**e**, **f**). The qRT-PCR assay described in Figs. 1 and 2 was used to estimate the total bacterial load and Rp load per tick *GAPDH*. The *p* value is provided to compare the statistical significance between the selenogene-silenced ticks and the control ticks. A *p* value of < 0.05 was considered statistically significant

Quantifying how the symbiont load changes in ticks may provide insight into potential pathogen–symbiont interactions inside ticks where selenogenes have been silenced. Therefore, FLE and CMM were quantified after selenogene knockdown in the tick tissues to assess how their levels might be altered by co-infection with *R. parkeri* (Fig. 7). In the *SELENOM* knockdown, the FLE levels were not significantly altered in the gut tissues (*p* = 0.35), but they were significantly reduced in the salivary glands (*p* = 0.0113). CMM loads in both midgut and salivary gland tissues were not affected by the *SELENOM* knockdown (*p* = 0.59 and *p* = 0.118, respectively). In contrast, the CMM levels were significantly depleted in the *SELENOO* knocked-down midgut tissues (*p* = 0.0042) (Fig. 7d) and in the salivary glands (*p* = 0.025) with the *SELENOS* knockdown (Fig. 7f).

## Discussion

**Fig. 8:**
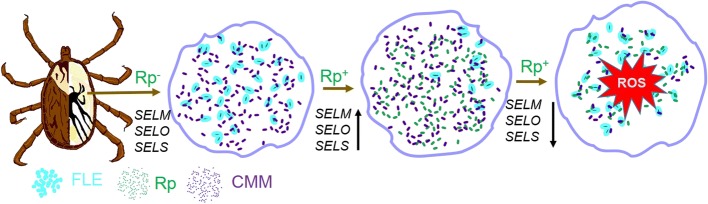
Proposed model for *Rickettsia*–symbiont interactions and the functional significance of selenoproteins in rickettsial replication inside tick tissues. The colonization of *R. parkeri* in *A. maculatum* gives it an opportunity to dynamically interact with tick symbionts and modulate tick defenses by regulating tick gene expression (e.g., selenogenes). In *R. parkeri-*free (Rp^−^) tick cells, FLE and CMM are present and normal expression levels of tick selenoproteins (*SELENOM*, *SELENOO*, and *SELENOS*) occur. In *R. parkeri-*infected (Rp^+^) tick cells, the normal symbiont dynamics are altered such that CMM replicates and FLE numbers decline, and the selenoprotein expression levels are upregulated. Knocking down the selenoproteins by RNA interference reduces the selenoprotein expression levels, and the elevated levels of reactive oxygen species impair the replication of both *R. parkeri* and CMM while FLE replicates at normal levels. *SELENOM (SELM)-SELENO (SELO)-SELENOS (SELS)*

Pathogen proliferation within tick tissues is an important aspect of the overall vector competence of ticks. This study investigated possible interactions between pathogenic *R. parkeri* and two non-pathogenic symbionts found in *A. maculatum* and whether selenoproteins might influence replication in *R. parkeri* and in the two tick symbionts, FLE and CMM (Fig. 8 and Table 1). In ticks, blood meal uptake can adversely affect bacterial replication because of the oxidative stress that is associated with it [10]; however, *R. parkeri* is able to defy oxidative stress and can multiply in the tick despite blood meal acquisition by the immature tick stages (Fig. 1a). The total bacterial load in Rp^+^ ticks compared with that in Rp^−^ ticks is possibly higher because of the additional rickettsial and symbiont load in the Rp^+^ ticks (Fig. 2) which, like with *Borrelia burgdorferi* in *I. scapularis*, is able to increase in the tick tissues even in the presence of the blood meal [41]. Both of the aforementioned studies lack detail about how many bacteria a tick inoculates into the host during feeding. In fact, they only considered the bacterial load residing in the tick tissues, particularly in the unfed and blood-fed stages (Figs. 1 and 2). Nevertheless, our results confirm that transovarial and transstadial maintenance of *R. parkeri*, FLE, and CMM occurs in *A. maculatum*, a finding reported previously for other tick species [42–44]. The genomic sequence of FLE from *A. maculatum* has been published [27], but no direct experiments have confirmed the specific role of this agent in the tick. But interestingly, an elegant study by Duron et al. [45] identified a *Francisella* type called F-Om, which they discovered was a “nutritional mutualist” involved in the synthesis of B vitamins in the in the African soft tick, *Ornithodoros moubata.* The role played by CMM in its host has not elucidated, but its presence may be related to the supply of important nutrients that are deficient in tick blood meals [46]. Microbial symbionts play crucial roles in arthropod physiology and pathogen colonization, and innovative use of these symbionts could offer a novel method for controlling vector-borne disease transmission [28]. The CMM microbes identified in one study have a unique intra-mitochondrial life cycle [43] and have been reported to occur in other blood feeding arthropods [47]. Interestingly, large CMM loads have been found in *I. holocyclus* [48], and these microbes are known to flourish in the presence of a blood meal in *I. ricinus* [49], but this was not the case here with *A. maculatum* (Additional file 1: Figure S1)*.* Rather, CMM in *A. maculatum* can be viewed as a successful colonizing partner of *R. parkeri*. Except in eggs and unfed larva, CMM loads were consistently higher in Rp^+^ ticks than in Rp^−^ tick tissues including the midguts, salivary glands, and ovaries from fed or unfed female adult ticks. Unlike CMM, FLE levels became reduced in *R. parkeri*-infected female tick midguts but not in the salivary glands, ovaries, eggs, nymphs, or larvae (Figs. 2 and 3). These results suggest a possible synergistic relationship between *R. parkeri* and CMM during their transstadial transmission. Cafiso et al. [50] detected and quantified CMM bacteria in seven tick species and hypothesized diverse roles of this bacteria in variety of different tick species. Clearly, more work is needed in this area, but our results provide the first evidence that FLE could be an important symbiont in ticks, which like *Wolbachia* or *Chromobacterium* (Csp_P) in mosquitoes are able to modulate the replication of the malaria parasite and dengue virus [51, 52]. The refractoriness of pathogen transmission to humans from the arthropod vector has been linked to the mosquito symbiont, *Wolbachia* [53]. The presence of FLE possibly interferes with *R. parkeri* replication in *A. maculatum*, which bears similarity to defensive symbiosis where the presence of one or more symbionts interferes with pathogenic bacteria colonization of the vector gut [53] but differs from nutritional symbiosis where the bacterial symbionts supplement the nutrients in the host [54, 55]. Here, based on the higher concentration of CMM that we found in the infected tick tissues and the reduced *R. parkeri* load, we believe that the presence of CMM favors the replication of *R. parkeri* replication (Figs. 2, 3, and 6). We also believe that CMM might have a synergistic effect on *R. parkeri*, and displacing this symbiont may also displace *R. parkeri*-infected ticks in the field, as was recently shown in a study on *Candidatus* Rickettsia andeanae [56].

We further investigated the dynamic interplay occurring among *R. parkeri,* CMM, and FLE microbes in ticks depleted of *R. parkeri* via selenoprotein silencing (Figs. 6 and 7). We found that infection with *R. parkeri* differentially regulated a battery of tick selenoproteins in the midgut, salivary glands, and ovarian tissues (Fig. 4). We showed the differential expression of selenogenes within and among the tick organs with respect to the pathogen infection. The selenoproteins in the salivary glands were highly expressed during infection with *R. parkeri* compared with those in the midguts and ovarian tissues (Fig. 4). Pathogen development and the secretory functions of the tick salivary glands might have resulted in the higher levels of selenoprotein expression in tick salivary glands that were observed. Furthermore, during tick feeding, the tick salivary glands probably remained under stress because these glands participate in the constant supply of anti-hemostatic, anti-inflammatory, and analgesic compounds during the continuous flow of the blood meal [57, 58]. ROS generation is one of the first lines of defense against invading microbes [59, 60]. However, despite minimal investigation, evidence is now accumulating that the tick selenoproteome and antioxidant enzymes may play critical roles in detoxifying ROS and in maintaining both vector microbiota and *R. parkeri* colonization [10, 31, 34, 36–38].

The compensatory actions of the redox genes following selenogene transcript depletion via RNAi differed between the Rp^−^ and Rp^+^ ticks (Fig. 5, Additional file 3: Figure S3). *SELENOM* depletion in the Rp^+^ ticks showed evidence of compensation by overexpression of *TrxR*, *Mn-SOD*, and *Salp25D* in the midgut and ovary tissues, whereas *CAT*, *SELENOK*, and *SELENOS* were only upregulated in the salivary glands (Fig. 5a). *Cu/Zn-SOD* and *Duox* were significantly upregulated after *SELENOO* and *SELENOS* silencing; these are involved in known defense mechanisms against invading pathogens and are also involved in repairing the tissue damage from *Rickettsia*-dependent superoxide generation [61]. Superoxide generation is associated with rickettsial infections [62], and tick extracellular *Cu/Zn-SOD* is the main quencher for dismutation of the superoxides generated during rickettsial infections [38]. Upregulated *Cu/Zn-SOD* probably provides the redox balance required to offset the superoxide radicals generated by the rickettsial infections in the ticks after *SELENOO* and *SELENOS* were knocked down, but *Cu/Zn-SOD* was not upregulated in the *SELENOM* knockdowns (Fig. 5). Further investigation of the unfolded protein response sensor genes (*ATF6* and *IRE1*) provided evidence of the altered protein folding homeostasis inside the ER, the organelle necessary for the proper folding of all secretory and transmembrane proteins (Additional file 4: Figure S4). The sensor genes for the unfolded protein response (*ATF6* and *IRE1*) and tick selenoprotein silencing potentially cause ER stress [63]. Knockdown of the mitochondrial resident selenogene, *SELENOO*, likely induced high oxidative stress in the gut tissues (Fig. 5, Additional file 4: Figure S4), which in turn induced ER stress. Studies have shown that there is a physical and biochemical interaction between the ER and mitochondria [64], and mitochondrial ROS can induce ER stress [65]. We proposed a model to summarize the important points arising from our study (Fig. 8). In this model, we suggest that successful *R. parkeri* replication within the tick vector is enhanced by the presence of CMM, probably by displacing FLE. The selenogenes responding to *R. parkeri* infection by transcriptional upregulation favor *R. parkeri* replication, and this in turn enhances the overall vectorial competence of *A. maculatum* for *R. parkeri*.

## Conclusion

The successful growth of a human pathogenic spotted fever group rickettsia, *R. parkeri*, inside its competent vector, *A. maculatum*, offers it a chance to dynamically interact with tick symbionts and modulate its host’s defenses by upregulating tick selenoproteins. This study illustrates the potential of a new research model aimed at providing better understanding of tick–pathogen interactions and the important roles played by symbionts and various tick factors in regulating pathogen growth.

## Methods

### Ethics statement

All the animal experiments were performed in strict accordance with the recommendations in the Guide for the Care and Use of Laboratory Animals of the National Institutes of Health, USA. The protocols for tick blood feeding were approved by the Institutional Animal Care and Use Committee of the University of Southern Mississippi (protocol#15101501 and 15011402).

### Ticks and tissue preparations

*A. maculatum* ticks were maintained at the University of Southern Mississippi according to established methods [66]. The Rp^+^ tick colonies and Rp^−^ tick colonies from Mississippi field collections were established and maintained in the laboratory. *A. maculatum* colonies containing individual Rp^+^ and Rp^−^ ticks were established in our laboratory in 2013. Questing unfed adult ticks were collected from Mississippi Sandhill Crane, National Wildlife Refuge, Gautier, Mississippi (www.fws.gov/refuge/mississippi_sandhill_crane/) using the drag cloth method during July and August 2013. The hundreds of ticks collected from the field were blood-fed on sheep and allowed to fully engorge and drop off. Fully engorged female adult ticks were kept in snap vials for egg laying. Typically, a single fully engorged female *A. maculatum* lays on average a 450–950 mg egg mass containing 15,000–18,000 eggs. To determine the presence of *R. parkeri* in an egg mass, 10–20 mg of it from each gravid female was subjected to genomic DNA extraction using the DNeasy extraction kit as described by the manufacturers (Qiagen, CA) followed by PCR amplification of the extracted DNA to identify SFGR species [9]. Individual Rp^+^ and Rp^−^ eggs were selected from individual gravid females and allowed to hatch into unfed larva. The unfed larval ticks were blood-fed by allowing them to infest golden Syrian hamsters until they reached repletion. Fully engorged larvae were allowed to molt into nymphs and then blood-fed on hamsters. Fully engorged nymphs molted as male or female ticks. Each developmental stage was routinely tested for the presence of *R. parkeri* infection*.* Closed colonies from the third and fourth generation of the original wild-caught ticks were used in this study after confirming the presence or absence of infection in the colonies. Freshly laid eggs, freshly molted larvae and blood-fed larvae, freshly hatched nymphs, and blood-fed nymphs were collected from Rp^+^ and Rp^−^ colonies separately. Eggs, unfed larvae (from three individual ticks, 20 mg each), three fed and pooled larval batches, unfed nymphs (20 mg), and fed nymphs were stored in RNAlater (Invitrogen, Carlsbad, CA). At least three biological replicates were used in all the experiments. Tick tissues from the unfed and partially blood-fed female adult ticks were dissected and stored immediately in RNAlater (Invitrogen) prior to extracting the mRNA using our previously described laboratory method [34].

### RNA preparation, cDNA synthesis, and qRT-PCR

Total RNA extraction, cDNA synthesis, and qRT-PCR were conducted as previously described [38]. The gene-specific primer sequences, which were designed to amplify specific cDNA fragments from *A. maculatum* tissues, are listed in Additional file 5: Table S1. Transcriptional gene expression of the tick genes in Rp^−^ ticks was normalized against *β-actin* gene expression, while *GAPDH* gene expression was used to normalize tick gene expression in the Rp^+^ tick tissues because it is stably expressed irrespective of the infection status [67]. The synthesized cDNA was used to measure mRNA levels by qRT-PCR using the CFX96 Real Time System (Bio-Rad Inc., Hercules, CA) as described previously [10, 38].

### Double-stranded RNA (dsRNA) synthesis, tick injections, and hematophagy

dsRNA was synthesized to allow for the in vivo analysis of *SELENOM*, *SELENOO*, and *SELENOS* in the ticks. Tick manipulations were performed according to the methods described previously [36, 68]. The dsRNAs for each selenoprotein gene (dsSELENOM, dsSELENOO, dsSELENOS) were diluted to working concentrations of 1 μg/μL in nuclease-free water. The same protocol was used to synthesize dsLacZ, which was used as an irrelevant dsRNA control. Twenty-five unfed adult female ticks were each microinjected with 1 μl of dsRNA or dsLacZ using a 27-gauge needle, then kept overnight at 37 °C to alleviate needle trauma and promote their survival, after which they were blood-fed using routine laboratory procedures [69].

### Quantification of total bacterial loads

The bacterial load in each tick tissue was estimated as described previously [10, 15]. The bacterial copy numbers were normalized against *A. maculatum* actin expression in the uninfected ticks and *GAPDH* expression in the Rp^+^ ticks.

### Quantification of FLE in tick tissues

The FLE from *A. maculatum* tick tissues was quantified using the primers described elsewhere [25]. The serially diluted copies (10^8^ to 10^1^) of each gene were PCR-amplified using predetermined thermal cycling conditions, and the Ct values for known dilutions were used to construct a standard curve from which the copy number of each gene was calculated. The 25 μL qRT-PCRs comprised 125 nM of each primer, SYBR Green Master Mix (Bio-Rad, Inc. USA), and the serially diluted PCR products prepared for each standard curve. The reaction mixtures were subjected to the thermal cycle parameters of 95 °C for 5 min followed by 29 cycles of 95 °C for 30s, 52 °C for 30s, and 72 °C for 30s with a final extension of 72 °C for 5 min in a CFX96 Real Time System (Bio-Rad Inc.). The FLE copy numbers were normalized against the *A. maculatum GAPDH* gene. As with the other qRT-PCRs, all the samples were run in triplicate.

### Quantification of CMM in tick tissues

We followed the protocol described previously for quantifying CMM [43]. The CMM-specific *GyrB* gene and tick *GAPDH* gene were PCR-amplified from *A. maculatum* ticks using the primers shown in Table 1. The amplified PCR products serially diluted tenfold (10^8^ to 10^1^ copies) were used to generate a standard curve. The qRT-PCRs comprised 400 nM of each primer and 25 ng of the cDNA samples. The reaction mixture containing SYBR Green (Bio-Rad Inc. USA) was subjected to thermal cycling at 95 °C for 2 min, 40 cycles at 95 °C for 15 s, and at 60 °C for 30s, and a melting curve from 55 °C to 95 °C with increasing increments of 0.5 °C per cycle was prepared using the CFX96 Real Time System (Bio-Rad Inc.). The standard curves generated were used to calculate the copy numbers of the CMM *GyrB* gene and the tick *GAPDH* gene. The CMM copy numbers were normalized against the *A. maculatum GAPDH* gene. As with the other qRT-PCRs, all the samples were run in triplicate.

### Quantification of the *R. parkeri* load in tick tissues

The level of infection with *R. parkeri* within the tick tissues across the developmental stages was quantified using a slightly modified version of a previously published method [9, 70]. The *R. parkeri* load was estimated as the ratio of *R. parkeri*-specific *rompB* gene copies to tick *GAPDH* copies. *GAPDH* and *rompB* genes were amplified using 250 nM of each specific primer (Table 1) in a reaction containing SYBR Green Master Mix (Bio-Rad Inc.) in the CFX96 Real Time System (Bio-Rad Inc.) with thermal cycling conditions of 95 °C for 10 min, followed by 35 cycles of 95 °C for 15 s, 60 °C for 30s, and 72 °C for 30s. The standard curves for tick *GAPDH* and *rompB* were prepared based on the amplification profiles of known concentrations of purified GAPDH and rompB PCR products. The standard curves generated were used to estimate the copy numbers of each gene in the tick samples.

### Quantification of total oxidative stress levels

The malondialdehyde lipid peroxidation assay kit (Sigma-Aldrich, St. Louis, MO, USA) was used to quantify lipid degradation as a result of oxidative damage [38]. All the procedures followed the manufacturer’s recommendations, and all the samples were balanced by weight.

### Data analysis

All data are expressed as mean values ± SEM unless otherwise indicated. Statistical significance between two experimental groups or their respective controls was determined by a *t* test (*p* value, 0.05). Comparative differences among multiple experimental groups were determined by analysis of variance with statically significant *p* values of < 0.05 (GraphPad Prism 6.05, La Jolla, CA). Transcriptional expression levels were determined using Bio-Rad software (Bio-Rad CFX MANAGER v.3.1), and the gene expression values obtained were considered statistically significant if a *p* value of 0.05 was obtained when compared with the control.

## Additional files

Additional file 1: **Figure S1.** FLE and CMM loads across the blood meal in naïve tick tissues. FLE loads in midguts (a) and salivary glands (b), and CMM loads in midguts (c) and salivary glands (d) at different time points in the tick tissues. (DOCX 117 kb)

Additional file 2: **Figure S2.** Time-dependent *SELENOO* (a) and *SELENOS* (b) transcriptional expression levels in uninfected (naïve) tick midguts and salivary gland tissues during the adult female blood meal. The change in transcriptional activity of (a) *SELENOO* and (b) *SELENOS* in *A. maculatum* midgut and salivary gland tissues was normalized to that of the unfed tick using *β-actin* as a reference gene. (DOCX 135 kb)

Additional file 3: **Figure S3.** Knockdown of tick selenoproteins in naïve (uninfected) *A. maculatum*. (a) ds*SELENOO* and (b) ds*SELENOS*. Gene expression in naïve ticks was normalized against tick *β-actin* as the reference gene. Compensatory antioxidant expression levels were measured for eukaryotic elongation factor (*eEFSec*), selenoproteins (*SELENOM*, *SELENOK*, *SELENOS*, *SELENOO*, *TrxR*, *SELENON*, *SELENOT*), mitochondrial superoxide dismutase (*Mn-SOD*), cytosolic superoxide dismutase (*Cu/Zn-SOD*), catalase (*Cat*), glutathione reductase (*GSHR*), and glutathione peroxidase (*Salp25D*). (DOCX 231 kb)

Additional file 4: **Figure S4.** The unfolded protein response. The unfolded protein response estimation for the salivary glands (SG) and midgut (MG) based on transcriptional gene expression of the *ATF6* and *IRE1* sensor genes when selenogenes *SELENOO* and *SELENOS* were silenced in Rp^−^ ticks (a) and Rp^*+*^ ticks (b). The effects of the *SELENOM* silenced tissues were also measured in the Rp^+^ ticks (b). (DOCX 177 kb)

Additional file 5: **Table S1.** Gene-specific PCR primers and probes used for qRT-PCR and RNAi studies. (DOCX 42 kb)
