# Supplementary material for: The tick endosymbiont Candidatus Midichloria mitochondrii and selenoproteins are essential for the growth of Rickettsia parkeri in the Gulf Coast tick vector
Source: Microbiome. 2018 Aug 13;6:141. doi: 10.1186/s40168-018-0524-2 (PMC6090677; doi:10.1186/s40168-018-0524-2)
Supplement: Supplementary file 1 — Figure S1. FLE and CMM loads across the blood meal in naïve tick tissues. FLE loads in midguts (a) and salivary glands (b), and CMM loads in midguts (c) and salivary glands (d) at different time points in the tick tissues. (DOCX 117 kb) [file 40168_2018_524_MOESM1_ESM.docx]

**Figure S1.** FLE and CMM loads across the blood meal in naïve tick tissues. FLE loads in midguts (a) and salivary glands (b), and CMM loads in midguts (c) and salivary glands (d) at different time points in the tick tissues.
