# Supplementary material for: The tick endosymbiont Candidatus Midichloria mitochondrii and selenoproteins are essential for the growth of Rickettsia parkeri in the Gulf Coast tick vector
Source: Microbiome. 2018 Aug 13;6:141. doi: 10.1186/s40168-018-0524-2 (PMC6090677; doi:10.1186/s40168-018-0524-2)
Supplement: Supplementary file 2 — Figure S2. Time-dependent SELENOO (a) and SELENOS (b) transcriptional expression levels in uninfected (naïve) tick midguts and salivary gland tissues during the adult female blood meal. The change in transcriptional activity of (a) SELENOO and (b) SELENOS in A. maculatum midgut and salivary gland tissues was normalized to that of the unfed tick using β-actin as a reference gene. (DOCX 135 kb) [file 40168_2018_524_MOESM2_ESM.docx]

**Figure S2.** Time-dependent *SELENOO* (a) and *SELENOS* (b) transcriptional expression levels in uninfected (naïve) tick midguts and salivary gland tissues during the adult female blood meal. The change in transcriptional activity of (a) *SELENOO* and (b) *SELENOS* in *A*. *maculatum* midgut and salivary gland tissues was normalized to that of the unfed tick using *β-actin* as a reference gene.
