# Supplementary material for: The tick endosymbiont Candidatus Midichloria mitochondrii and selenoproteins are essential for the growth of Rickettsia parkeri in the Gulf Coast tick vector
Source: Microbiome. 2018 Aug 13;6:141. doi: 10.1186/s40168-018-0524-2 (PMC6090677; doi:10.1186/s40168-018-0524-2)
Supplement: Supplementary file 3 — Figure S3. Knockdown of tick selenoproteins in naïve (uninfected) A. maculatum. (a) dsSELENOO and (b) dsSELENOS. Gene expression in naïve ticks was normalized against tick β-actin as the reference gene. Compensatory antioxidant expression levels were measured for eukaryotic elongation factor (eEFSec), selenoproteins (SELENOM, SELENOK, SELENOS, SELENOO, TrxR, SELENON, SELENOT), mitochondrial superoxide dismutase (Mn-SOD), cytosolic superoxide dismutase (Cu/Zn-SOD), catalase (Cat), glutathione reductase (GSHR), and glutathione peroxidase (Salp25D). (DOCX 231 kb) [file 40168_2018_524_MOESM3_ESM.docx]

**Figure S3.** Knockdown of tick selenoproteins in naïve (uninfected) *A. maculatum*. (a) ds*SELENOO* and (b) ds*SELENOS*. Gene expression in naïve ticks was normalized against tick *β-actin* as the reference gene. Compensatory antioxidant expression levels were measured for eukaryotic elongation factor (*eEFSec*), selenoproteins (*SELENOM*, *SELENOK*, *SELENOS*, *SELENOO*, *TrxR*, *SELENON*, *SELENOT*), mitochondrial superoxide dismutase (*Mn-SOD*), cytosolic superoxide dismutase (*Cu/Zn-SOD*), catalase (*Cat*), glutathione reductase (*GSHR*), and glutathione peroxidase (*Salp25D*).
