# Supplementary material for: The tick endosymbiont Candidatus Midichloria mitochondrii and selenoproteins are essential for the growth of Rickettsia parkeri in the Gulf Coast tick vector
Source: Microbiome. 2018 Aug 13;6:141. doi: 10.1186/s40168-018-0524-2 (PMC6090677; doi:10.1186/s40168-018-0524-2)
Supplement: Supplementary file 4 — Figure S4. The unfolded protein response. The unfolded protein response estimation for the salivary glands (SG) and midgut (MG) based on transcriptional gene expression of the ATF6 and IRE1 sensor genes when selenogenes SELENOO and SELENOS were silenced in Rp− ticks (a) and Rp+ ticks (b). The effects of the SELENOM silenced tissues were also measured in the Rp+ ticks (b). (DOCX 177 kb) [file 40168_2018_524_MOESM4_ESM.docx]

**Figure S4. The unfolded protein response.** The unfolded protein response estimation for the salivary glands (SG) and midgut (MG) based on transcriptional gene expression of the *ATF6* and *IRE1* sensor genes when selenogenes *SELENOO* and *SELENOS* were silenced in Rp^-^ ticks (a) and Rp*^+^* ticks (b). The effects of the *SELENOM* silenced tissues were also measured in the Rp^+^ ticks (b).
