# Supplementary material for: The tick endosymbiont Candidatus Midichloria mitochondrii and selenoproteins are essential for the growth of Rickettsia parkeri in the Gulf Coast tick vector
Source: Microbiome. 2018 Aug 13;6:141. doi: 10.1186/s40168-018-0524-2 (PMC6090677; doi:10.1186/s40168-018-0524-2)
Supplement: Supplementary file 5 — Table S1. Gene-specific PCR primers and probes used for qRT-PCR and RNAi studies. (DOCX 42 kb) [file 40168_2018_524_MOESM5_ESM.docx]

**Table S1. Gene-specific PCR primers and probes used for qRT-PCR and RNAi studies.**

| Gene | GenBank ID | Forward Primer (5'-3') | Reverse Primer (5'-3') | Size (bp) |
| --- | --- | --- | --- | --- |
| *Actin* | JO842238 | TGGCTCCTTCCACCATGAAGATCA | TAGAAGCACTTGCGGTGCACAATG | 169 |
| *Catalase* | JO843741 | AAAGGACGTCGACATGTTCTGGGA | ACTTGCAGTAGACTGCCTCGTTGT | 173 |
| *GPx / Salp25D* | JO843645 | TGCCGCGCTGTCTTTATTATTGGC | AGTTGCACGGAGAACCTCATCGAA | 102 |
| *GSHR* | JO844062 | ACCTGACCAAGAGCAACGTTGAGA | ATCGCTTGTGATGCCAAACTCTGC | 170 |
| *eEFSec* | KC989559 | TGGCTCCAGAAATGCTGCTCATTG | ACGCCTTTGCGACTCTTCTCCTTA | 157 |
| *SelK* | JO843326 | AGTTCCAGCAGGTCATCAGTGTCA | TCCAGGAATAGGGCAGTCCATTGT | 132 |
| *SelM (dsRNA)* | JO842653 | GACCTGATATGCTCGTCATGTAG | ATAGTGTCCGTGTCCCATTTC | 317 |
| *SelM (qRT-PCR)* | JO842653 | GCTGTACGATGAGAATGGAGAG | AGCCAGGTGCTCAAACAA | 94 |
| *SelN* | KC989560 | TTAGTTTGGACACTGTGGACGGGT | AGGCTTCTCTAACAACGGCACTCA | 150 |
| *SelO (dsRNA)* | KC989561 | GTTGGGCTCACCATTGACTA | CGTCTCCTCCATAGCATCATAAA | 321 |
| *SelO* | KC989561 | AAGCTCGGCCTTGTGAAGAGAGAA | TACAGCACGACAAGAGCTTGGACA | 190 |
| *SelS (dsRNA)* | JO842687 | CTAGCTTCGCTGATACAGTTCTC | TGGTTGACACACCTCCTTTC | 508 |
| *SelS* | JO842687 | AGAACAAGTGCACCACAACAGCAG | ATTTCTTGCATCCTTCGACGTGCC | 107 |
| *SelT* | KC989562 | TCTTTGTGTGTGGAGCCATCGAGA | ACCACACCCGCACGTCATTAAAGT | 81 |
| *SelX* | JO845128 | ACCACTCTCCTTGGCCATCATTCA | TGCACTTCCCACAGTACACCTTGA | 108 |
| *Cu-Zn SOD/SOD1* | JO844140 | GGAACCGAAGACAGCAAGAA | GAGAAGAGGCCGATGACAAA | 143 |
| *Mn- SOD/SOD3* | JO843979 | GCATCTACTGGAC AAACCTCTC | GCAGACATCAGGCCTTTGA | 115 |
| *Duox* |  | ATG ACG CAC AGC CTG TAT ATT | TGT CCA GAG TGA AGA CGA TTG | 123 |
| *TrxR* | JO843723 | TGTGACTACACCAACGTGCCTACA | AGTAGCCTGCATCCGTTCCTCTTT | 175 |
| *Caspase1* | JO842755 | GAGGAGTCTAGCAGGATGTTTC | ACTGTCATGCTCCGTGTAATC | 127 |
| *Caspase2* | JO845022 | GGTGATCGTGATGTCCTGTATG | CGACAGGCCTGAATGAAGAA | 128 |
| *GyrB* | AM159536 | CTTGAGAGCAGAACCACCTA | CAAGCTCTGCCGAAATATCTT | 146 |
| *rOmpB* | AF123717 | CAAATGTTGCAGTTCCTCTAAATG | AAAACAAACCGTTAAAACTACCG | 96 |
| *16S rRNA* |  | AGAGTTTGATCCTGGCTCAG | CATGCTGCCTCCCGTAGGAGT |  |
| FRAN*16S rRNA* |  | CAACATTCTGGACCGAT | TGCGGGACTTAACCCAACAT | 373 |
| *ATF6* |  | GGTTGTTGTACCACCCAAGA | GAAGTTGGCTGCAGGTATGA | 106 |
| *IRE1* |  | GTCATCCTCCTCATCAGACAA | GTGGTTCAACAATGGCAAGG | 107 |
| GAPDH | JO842341 | CACCCATCACAAACATGGGTGCAT | TTTCAGGAAATGAAGCCTGCCAGC | 175 |
